# Do genetic factors protect for early onset lung cancer? A case control study before the age of 50 years

**DOI:** 10.1186/1471-2407-8-60

**Published:** 2008-02-25

**Authors:** Albert Rosenberger, Thomas Illig, Katrin Korb, Norman Klopp, Vera Zietemann, Gabi Wölke, Eckart Meese, Gerhard Sybrecht, Florian Kronenberg, Matthias Cebulla, Maria Degen, Peter Drings, Andreas Gröschel, Nikolaus Konietzko, Karsten grosse Kreymborg, Karl Häußinger, Gerd Höffken, Bettina Jilge, You-Dschun Ko, Harald Morr, Christine Schmidt, E-Wilhelm Schmidt, Dagmar Täuscher, Heike Bickeböller, H-Erich Wichmann

**Affiliations:** 1 Department of Genetic Epidemiology, Georg-August University of Göttingen, Medical School, Germany; 2 Institute of Epidemiology, GSF-National Research Centre for Environment and Health, Germany; 3 Institute of Human Genetics, University of the Saarland, Germany; 4 Institute of Internal Medicine V, Medical School, University of the Saarland, Germany; 5 Division of Genetic Epidemiology, Department of Medical Genetics, Molecular and Clinical Pharmacology, Innsbruck Medical University, Austria; 6 Municipal Hospital Sankt Georg, Leipzig, Germany; 7 Clinic of Pneumology, Waldhof Elgershausen, Greifenstein, Germany; 8 Department of Oncology/Internal Medicine, Thoraxklinic of the Univeristy Hospital Heidelberg GmbH, Heidelberg, Germany; 9 Department of Pneumology, University Hospital Essen, Ruhrlandklinik, Germany; 10 Centre for Internal Medicine, University Hospital Leipzig, Germany; 11 Clinic of Pneumology München-Gauting, Munich, Germany; 12 Clinic Coswig GmbH, Division Internal Medicine, Coswig, Germany; 13 Chemnitz Hospital, Clinic for Internal Medicine, Chemnitz, Germany; 14 Department of Oncology, University Hospital Bonn, Germany; 15 Wald-Hospital Gera GmbH, II. Medical Clinic, Gera, Germany; 16 Institute of Medical Data Management, Biometry and Epidemiology, Ludwig Maximilians University Munich, Chair of Epidemiology, Germany

## Abstract

**Background:**

Early onset lung cancer shows some familial aggregation, pointing to a genetic predisposition. This study was set up to investigate the role of candidate genes in the susceptibility to lung cancer patients younger than 51 years at diagnosis.

**Methods:**

246 patients with a primary, histologically or cytologically confirmed neoplasm, recruited from 2000 to 2003 in major lung clinics across Germany, were matched to 223 unrelated healthy controls. 11 single nucleotide polymorphisms of genes with reported associations to lung cancer have been genotyped.

**Results:**

Genetic associations or gene-smoking interactions was found for *GPX1(Pro200Leu) *and *EPHX1(His113Tyr)*. Carriers of the Leu-allele of *GPX1(Pro200Leu) *showed a significant risk reduction of OR = 0.6 (95% CI: 0.4–0.8, p = 0.002) in general and of OR = 0.3 (95% CI:0.1–0.8, p = 0.012) within heavy smokers. We could also find a risk decreasing genetic effect for His-carriers of *EPHX1(His113Tyr) *for moderate smokers (OR = 0.2, 95% CI:0.1–0.7, p = 0.012). Considered both variants together, a monotone decrease of the OR was found for smokers (OR of 0.20; 95% CI: 0.07–0.60) for each protective allele.

**Conclusion:**

Smoking is the most important risk factor for young lung cancer patients. However, this study provides some support for the T-Allel of *GPX1(Pro200Leu) *and the C-Allele of *EPHX1(His113Tyr) *to play a protective role in early onset lung cancer susceptibility.

## Background

Lung cancer is the most common cause of death from cancer in the world. The estimated total number of cases is 1.2 million annually and is still increasing [1,2]. For men lung cancer mortality is declining in Germany since nearly a decade, whereas the incidence in women is increasing. However for men and women of age 50 or younger the incidence of lung cancer is low [1].

The major cause of lung cancer is tobacco smoke, primarily of cigarettes, increasing the risk 15- to 30-fold. 90% of lung cancer cases can be attributed to a life long inhalation of tobacco smoke [1,3]. Additionally occupational (e.g. asbestos at workplace), environmental (e.g. passive smoking or ambient air pollution) and behavioral risk factors (e.g. diet) have been identified [1,2,4-14].

The median age of onset is 66 years; about 5% to 10% of patients are younger than 50 years. These young patients differ from older patients regarding the distribution of sex, histological type of the neoplasm and in genetic susceptibility [15-24]. Smoking remains to be the major risk factor in these younger patients [25-27], but familial aggregation of lung cancer was identified as a consistent additional risk factor in several epidemiological studies [28-37]. Recent investigations from Germany showed a 2.6-fold increased lung cancer risk in young patients (OR, 95% CI 1.6–6.0) if first degree relatives had cancer[27] and a 4.7-fold increased risk if a parent or sibling was affected with lung cancer [38]. Even for nonsmokers in the age between 40 and 59 an increase of the lung cancer risk up to 6-fold was seen in the presence of lung cancer in a first-degree relative [39].

The results of a segregation analysis suggest the presence of a high risk gene contributing to early-onset of lung cancer particularly in nonsmokers [40]. Another indication for a genetic contribution to lung cancer in the young is given by a larger increase of risk in monozygotic compared to dizygotic young twins, which was more evident in female than in male twins [41]. No such risk differences could be seen in a cohort of twins older than 50 [42]. Hence, the etiology of lung cancer in patients before age 50 seems to differ from that in older patients by a stronger genetic component, likely to interact with the exposure to tobacco smoke.

While smoking the body absorbs numerous carcinogens that need to be eliminated. In recent years several cytogenetic and molecular biological studies indicated chromosomal regions or candidate genes as linked to or associated with lung cancer. For example, a major susceptibility gene locus in the region of 6p23-25 was found to be linked in families with three or more individuals affected by lung, throat, or laryngeal cancer [43]. It can be hypothesized that gene products regulating phase I and II enzymes [15,44,45], tumor suppressor genes [46] and DNA repair genes [47-49] are associated with the development of lung cancer, but results are contradictory.

This study was set up to further clarify associations of DNA variants in candidate genes for metabolizing enzymes, a tumor suppressor gene and genes relevant for DNA repair and their interaction with smoking in lung cancer patients with age of onset 50 years or younger.

## Methods

### Study Design and Study Subjects

We carried out a frequency matched case-control study.

Caucasian patients with newly diagnosed and histologically or cytologically confirmed primary lung cancer with age 50 years or younger were recruited in 21 major lung clinics across Germany. They completed an interviewer-administered questionnaire in which detailed information on personal history, history of lung diseases, family history of cancer and smoking habits were assessed. Blood samples were taken from all patients. A DNA bank was established. From July 2000 to April 2003 blood samples and case report forms were obtained from 247 young lung cancer patients. One of the patients was excluded because the parents were Vietnamese.

Cancer free control individuals are a random sample (frequency matched by 5-year age categories and sex to cases) drawn from the participants of a population based survey (KORA – Cooperative Health Research in the Region of Augsburg [50], survey S4). KORA, a continuation of the WHO MONICA study, provides a platform for research in epidemiology, health economics and genetics, where data and blood samples can made available. Since 1984/85 four representative surveys have been performed, including approximately 4000 – 5000 adults each.

After excluding one control individual because the preparation of the blood sample for genotyping failed, a total of 246 cases were subsequently compared with 223 control individuals. With respect to yet genotyped markers neither major population stratification between the whole KORA sample (southwest Germany) or two other cohorts from Northern Germany [51] nor deviations in the Minor Allele Frequencies (MAFs) to those of the HapMap Ceu Population could be detected.

The study was approved by the ethics committee of the Bayerische Landesärztekammer München and all necessary local ethic committees of the involved recruitment clinics. All participants signed an informed consent form.

### Selection of candidate genes and single nucleotide polymorphism (SNP)

The selection of DNA variants in candidate genes for this study was based on two criteria: published significant association together with plausible biological relevance of a polymorphism to lung cancer. We searched MEDLINE for reviews about the genetics of lung cancer published between 1995 and 2002. (Search term: "Lung Neoplasms/genetics" [Mesh] AND ("molecular" [TI] OR "gene" [TI] OR "genetic" [TI]) Limits: Publication Date from 1995/1/1 to 2003/1/1, Humans, Review, English, German). From 138 hits, we selected 35 by screening for promising titles or abstracts. All mentioned genes and DNA variants with significant association in these reviews and in a wide ranging selection of original study reports were listed. Two experts in the molecular biology of cancer rated these DNA variants for plausible biological relevance to lung cancer. The final selection furthermore also needed to meet limited financial constraints.

We investigated the following 11 SNPs: *CYP1A1(Val462Ile) *(Cytochrom-P450 Enzyme, rs1048943), *EPHX1(His113Tyr) *and *EPHX1(Arg139His) *(microsomal Expoide Hydrolase, rs1051740, rs2234922), *GSTP1(A-193C) *(Glutathione S-Transferase, rs947895), *NAT2(Thr114Ile) *and *NAT2(Gln197Arg) *(N-Acetyl-Transferase 2, rs1801280, rs1799930), *GPX1(Pro200Leu) *(Glutathione Peroxidase 1, rs1050450), *p53(Arg72Pro) *(tumor suppressor gene, rs1042522), *XRCC1(Arg280His) *and *XRCC1(Arg399Gln) *(X-ray Repair Cross-Complementing group1, rs25489, rs25487) and finally *XPD(Asp312Asn) *(Xeroderma Pigmentosum group D, rs1799793).

### Blood Sampling and Genotyping

Blood samples (4 × 9 ml) were taken by clinicians and sent to the study center (GSF-National Research Centre for Environment and Health) within 24 hours. Immortalized cell lines were prepared and stored in liquid nitrogen. DNA was isolated from fresh or frozen blood using the DNA isolation kit of Gentra, Minneapolis, and stored at -80°C. Genotyping of SNPs was performed by matrix-assisted laser desorption/ionization time-of flight (MALDI-TOF MS, Sequenom) according to Weidinger et al. [50]. Standard genotyping quality control including 10% duplicate samples, checking for Hardy-Weinberg equilibrium as well as negative samples revealed no major errors. In none of the duplicate samples a deviating genotype could be determined.

### Statistical Methods

When investigating potential modifications for lung cancer risk by marker genotypes, we considered sex, age and smoking habits as covariates. Patient's age was defined by age at first diagnosis, while for KORAS4 controls age at recruitment was recorded. Cumulative smoking exposure of former and current smokers was measured as packyears (PY). Cases and controls were grouped according to their smoking exposure level (SEL) into never and light smokers (≤1 PY), moderate (1-<20 PY) and heavy smokers (20 and more PY).

For cases we collected the smoking history in detail, as recommended [52]. For controls PY had to be approximated from the last amount of cigarette consumption per day and the duration of smoking. Preliminarily we classified all cases into the upper mentioned grouped by both concepts. We found these classifications to agree for 79% of cases. A similar agreement had been found by Bernaards et al. [53], when comparing retrospectively calculated PY with prospectively calculated PY. They concluded, that misclassification error in categorizing PY is smaller than quantitative error on continuous retrospectively PY calculation. Hence, we assume the use of PY groups to be at least as reliable as the collected retrospectively PY.

Using exact tests the distribution of histological subtypes was compared to a published German collection of 251 lung cancer patients with age of onset before the age of 46 years [20].

Hardy Weinberg equilibrium (HWE) was tested in controls using a likelihood ratio test [54].

As exact tests of genetic association we performed a BWS-Test (Baumgartner-Weiss-Schindler-Test [55]). These tests were also carried out in subsamples according to sex, age (grouped in age of onset ≤ 45 years and age of onset = 46–50 years, which almost splits the sample in two equally sized groups), smoking status (never, former and current smoker) as well as SEL (never and light, moderate, heavy smokers) and histological tumor subtype (small-cell, SCC and adenocarcinoma). For markers showing any significant association we performed two logistic regression models including age and sex as covariables. In model I smoking exposure level (SEL) was incorporated as the main effect while the genotype was nested within the SEL groups. Thus the genetic association was investigated nested within the SEL groups. The relative chance for lung cancer is estimated compared to genetic protected of the same smoking exposure. We also test for modification of the genetic effect by smoking by testing the contrast between the estimated parameters of model I for never or light smokers versus for moderate or heavy smokers.

In model II SEL-genotype interaction was directly included. Here all effects are given in comparison to wildtype-never and lightsmokers. The relative chance for lung cancer is estimated compared to genetic protected never or light smokers. Similar models were fitted with smoking status instead of SEL.

When appropriate, only subgroups of patients of a particular histological subtype were included. Motivated by single marker results on two genes we defined a genetic protection score (gPS) as the count of protective alleles, which are the T-allele of *GPX1(Pro200Leu) *and the C-allele of *EPHX1(His113Tyr)*. Logistic regression including gPS was performed as described above.

We also carried out a sensitivity analysis for missing data. The level of significance was set to 5% for all tests. To take multiple comparisons into account, the p-values of BWS tests were interpreted at the familywise significance level of 5%/11 = 0.445%.

## Results

Most patients were men (75%). The median age at diagnosis was 46 years for both sexes, which ranges from 24 to 50 years. For about 80% of the cases both parents were originating from Germany, further 8% had at least one German parent. Almost all non-German parents came from other European countries or North-America. Table 1 shows the characteristics of patients and controls.

**Table 1:** Sex, age smoking (in pack years) and histological subtypes of lung cancer in the study samples

	Cases		Controls	
**Sample size**	246		223	
**Sex **(m/f)	185/61 (~3:1)		167/56 (~3:1)	
	Male	Female	Male	Female
**Age **(mean +/- sd)	45.5 ± 4.1	44.9 ± 4.9	44.8 ± 3.9	44.9 ± 3.3
24–40 years	21 (11%)	7 (11%)	19 (11%)	6 (11%)
41–45 years	57 (31%)	18 (30%)	61 (37%)	24 (43%)
46–50 years	107 (58%)	36 (59%)	87 (52%)	26 (46%)
**Smoking**				
Packyears (mean +/- sd)*	32.3 ± 17.5	26.2 ± 12.4	27.1 ± 13.3	14.6 ± 10.2
never and light smoker (≤1 PY)	7 (4%)	8 (14%)	66 (65%)	32 (73%)
moderate smoker (1-<20 PY)	40 (23%)	14 (24%)	12 (12%)	9 (20%)
heavy smoker (20-PY)	130 (73%)	37 (63%)	23 (23%)	3 (7%)
former smoker	4	--	66	12
**Histological subtype**				
Small-cell (SCLC)	45 (24%)	20 (33%)		
Adenocarcinoma	54 (29%)	23 (37%)		
Squamous-cell carcinoma (SCC)	55 (30%)	9 (15%)		
Others	31 (17%)	9 (15%)		
Total	185	61		

* for controls packyears (py) have been calculated from the current amount of cigarette consumption per day and the duration of smoking

### Histological subtypes of lung cancer

As expected, the leading histological subtypes were squamous-cell carcinoma (SCC) in men (30%) and adenomacarcinoma in women (37%). The gender specific distributions of histological subtypes within cases were similar to an other German study [20]. For more details see Table 1.

### Smoking habits

Nearly all patients (97% of men, 87% of women) were ever smokers (current or former) with high tobacco consumption (current smokers: mean 32.4 PY, former smokers: mean 28.8 PY). According to the cumulative smoking dose 2 of 3 patients (men: 73%, women: 63%) were classified as highly exposed to tobacco (≥20 pack years), while in controls this were 14% and 5%, respectively. Furthermore, the frequency of 55% female smokers among patients clearly exceeded the nationwide percentage given by the micro-census 1998 (31% at 15 to 50 years of age) [56].

### Genotypes

The call rates of genotyping were on average 91% across markers.

Estimated allele frequencies are given in Table 2. Significant departures from HWE were not found in controls and only for p53 (Arg72Pro) in patients (p = 0.0384). Results of BWS-tests for a genetic association, an estimator of a main genetic effect within the total study population, age groups, male and female and current smokers are given in Table 3.

**Table 2:** SNP allele frequencies compared with frequencies from literature

Marker	Minor Allele	Group*	Call rate	Genotype Frequency [%]^§^				Allele Frequency [%]		
				
				n	0	1	2		95% CI	pub.**
*CYP1A1(VAL462ILE)*	G	P	91%	217	93	7	0	3	3–4	2 [57]
		C		212	92	8	0	4	3–4	
*GSTP1(A-193C)*	G	P	94%	221	43	45	12	34	32–36	30 [58]
		C		218	43	49	8	32	30–34	
*EPHX1(His113Tyr)*	C	P	96%	233	55	38	7	26	25–28	32 [57]
		C		219	49	43	8	29	28–31	
*EPHX1(Arg139His)*	G	P	86%	191	68	29	4	18	17–20	22 [57]
		C		208	69	27	3	17	16–18	
*NAT2(Thr114Ile)*	C	P	94%	222	34	51	15	41	39–43	46 [57]
		C		218	31	55	14	42	39–44	
*NAT2(Gln197Arg)*	A	P	95%	229	50	44	7	28	27–30	29 [57]
		C		218	50	41	10	30	28–32	
*XPD(Asp312Asn)*	A	P	92%	221	39	45	15	38	36–40	33 [49]
		C		209	37	45	18	40	38–42	
*XRCC1(Arg280His)*	A	P	94%	232	91	9	1	5	5–6	3 [59]
		C		211	90	10	0	5	5–6	
*XRCC1(Arg399Gln)*	A	P	86%	197	43	44	13	35	33–38	38 [59]
		C		204	43	45	12	34	32–37	
*GPX1(Pro200Leu)*	T	P	85%	186	61	34	5	22	20–24	31 [60]
		C		207	47	43	10	31	29–33	
*p53(Arg72Pro)*^§§^	C	P	87%	201	56	34	10	27	25–29	33 [61]
		C		204	56	38	6	25	23–27	

* P: patients, C: controls

** published allele frequency within Caucasian populations [reference]

§ 0. homozygous wildtype allele, 1. heterozygous, 2. homozygous for predisposing allele

§§ Only the distribution of genotypes of p53(Arg72Pro) in cases (P) differed significantly from HWE (p = 0.0384, likelihood ratio test)

**Table 3:** p-values for Baumgartner-Weiss-Schindler test for genetic association and OR for main genetic effects

Marker					Sex		Current smoker
						
		Total study pop.			Women	Men	
		
		p-value	OR**	95%-CI	p-values		
*CYP1A1(Val462Ile)*	rs1048943	0.857	0.68	0.30–1.51	1.000	0.838	0.833
*GSTP1(A-193C)*	rs947895	0.213	0.96	0.62–1.48	0.396	0.138	0.166
*EPHX1(His113Tyr)*	rs1051740	0.175	0.67	0.44–1.03	0.178	0.419	**0.015**
*EPHX1(Arg139His)*	rs2234922	0.366	1.41	0.86–2.30	0.243	0.475	0.342
*NAT2(Thr114Ile)*	rs1801280	0.445	1.01	0.64–1.59	0.450	0.355	0.131
*NAT2(Gln197Arg)*	rs1799930	0.252	1.02	0.66–1.55	0.241	0.445	0.291
*XPD(Asp312Asn)*	rs1799793	0.262	0.96	0.61–1.50	0.437	0.155	0.388
*XRCC1(Arg280His)*	rs25489	0.417	0.76	0.38–1.50	0.354	0.793	0.624
*XRCC1(Arg399Gln)*	rs25487	0.385	0.82	0.52–1.29	0.409	0.409	0.453
*GPX1(Pro200Leu)*	rs1050450	0.002*	0.50	0.32–0.79	**0.020**	**0.011**	**0.034**
*p53(Arg72Pro)*	rs1042522	0.190	1.14	0.72–1.80	0.127	0.385	0.278

p-values < 0.05 are bold.

* p-values below Bonferroni-corrected significance level of α' = 0.05/11 = 0.0045

** OR: Odds ratios adjusted for smoking exposure level (SEL), age and sex

### Genetic association analysis

The estimated odds ratios for lung cancer were OR = 6.6 (95% CI: 3.4–12.8) for moderate and OR = 22.7 (95% CI: 11.9–43.3) for heavy smokers without taking any genetic marker information into account.

### GPX1(Pro200Leu)

Among the 11 markers investigated, only the marker for the ***GPX1(Pro200Leu) ***gene showed a significant difference in the distribution of genotypes between all cases and controls (p_BWS-Test _= 0.002). The variant T-allele was associated with a lower risk for lung cancer and showed a frequency of 22% (95% CI: 20%–24%) in cases, compared to 31% (95% CI: 29%–33%) in controls. Significant association could also be observed within men (p_BWS-Test _= 0.011) and women (p_BWS-Test _= 0.020), current (p_BWS-Test _= 0.034), former smokers (p_BWS-Test _= 0.001) and heavy smokers (p_BWS-Test _= 0.003), in patients younger than 46 years of age (p_BWS-Test _= 0.004), in patients with adenocarcinoma (p_BWS-Test _= 0.024) and in patients with SCC (p_BWS-Test _= 0.007).

Carriers of the T-allele showed a risk reduction of OR = 0.3 (95% CI:0.1–0.8, p = 0.012) within heavy smokers whereas for moderate smokers (OR = 0.6, 95% CI:0.2–1.6, p = 0.278) and for never and light smokers (OR = 0.9, 95% CI:0.3–3.0, p = 0.825) significance was not reached. Because of the small number of never or light smokers beyond cases no significance (p = 0.9012) was achieved when testing for modification or the genetic effect by SEL groups. Even if the T-allele seems to have some protective effect, the risk for lung cancer for moderate and heavy smokers in the presence of a T-allele is clearly increased compared to 'genetically unprotected' never and light smokers (moderate: OR = 10.1, heavy: OR = 22.1). For more details see Table 4.

**Table 4:** Genetic association estimates and smoking for *GPX1(Pro200Leu) and EPHX1(His113Tyr)*

			Relative chance for LC compared to ...			
			Genetic protected never or light smokers *		Genetic protected of the same smoking exposure*.^§^	

SEL: smoking exposure level	Genetic disposition	n cases:controls	OR	*95% CI*	OR	95% CI

***GPX1(Pro200Leu)***						
Never and light smoker	CC	11:53	***1.0***	***Reference***	***1.0***	***Reference***
	T-carrier	5:42	0.9	0.3 – 3.0	0.9	0.3 – 3.0
Moderate smoker	CC	30:9	18.4	6.1 – 55.8	***1.0***	***Reference***
	T-carrier	15:12	10.1	3.4 – 30.2	0.6	0.2 – 1.6
Heavy smoker	CC	86:9	70.1	24.5 – 200	***1.0***	***Reference***
	T-carrier	52:16	22.1	8.3 – 59.3	0.3	0.1 – 0.8

***EPHX1(His113Tyr)***						
Never and light smoker	TT	1:10	***1.0***	***Reference***	***1.0***	***Reference***
	C-carrier	18:88	1.4	0.5 – 4.1	1.4	0.5 – 4.1
Moderate smoker	TT	4:2	40.0	12.2 – 131	***1.0***	***Reference***
	C-carrier	58:19	11.1	4.0 – 31.2	0.2	0.1 – 0.7
Heavy smoker	TT	14:2	58.2	20.9 – 161	***1.0***	***Reference***
	C-carrier	153:23	45.5	16.6 – 124	0.8	0.3 – 1.9

* odds ratio. adjusted for sex and age

^§ ^odds ratio of allele carriers compared to non carriers within the same smoking group

SEL smoking exposure level: never and light smokers: ≤1 pack years. moderate smokers: 1-<20 pack years. heavysmokers: ≥20 pack years.

### EPHX1(His113Tyr)

The SNP within exon 3 of the ***EPHX1 *gene **showed significant associations within current smokers (p_BWS-Test _= 0.015). Within current smokers the variant C-allele was associated with a lower risk for lung cancer and showed a frequency of 25% (95% CI: 21%–28%) for cases, compared to 36% (95% CI: 32%–40%) for controls.

We could find a risk decreasing genetic effect for C-carriers only for moderate smokers (OR = 0.2, 95% CI:0.1–0.7, p = 0.012). Please note the small number of TT-carriers within cases and controls, which lowers the evidence – not the significance – of this finding. No such significant effect was found for heavy smokers (OR = 0.8, 95% CI:0.3–1.9, p = 0.593), where the 95%-confidence interval for OR does not cover the point estimate of OR for moderate smokers. For more details see Table 4. Because of the small number of never or light smokers beyond cases no significance (p = 0.1898) was achieved when testing for modification or the genetic effect by SEL groups.

### GPX1(Pro200Leu) *and *EPHX1(His113Tyr)

As a combined effect of these two polymorphisms, one might look at the count of protective alleles (T for *GPX1(Pro200Leu)*, C for *EPHX1(His113Tyr)*) as a genetic prediction score (gPS). Such a score – modeled as continuous variables – yields an estimated OR per predisposing allele of 1.03 (95% CI: 0.5–2.0, p = 0.943) for never and light smokers, OR = 0.48 (95% CI: 0.2–1.1, p = 0.067) for moderate and OR = 0.55 (95% CI: 0.3–0.9, p = 0.020) for heavy smokers (over all SEL levels: p = 0.033). The difference between moderate and heavy smokers was not found significant (p = 0.777). Because of the small number of never or light smokers beyond cases no significance (p = 0.1770) was achieved when testing for modification or the effect of gPS by SEL groups.

**Figure 1:**
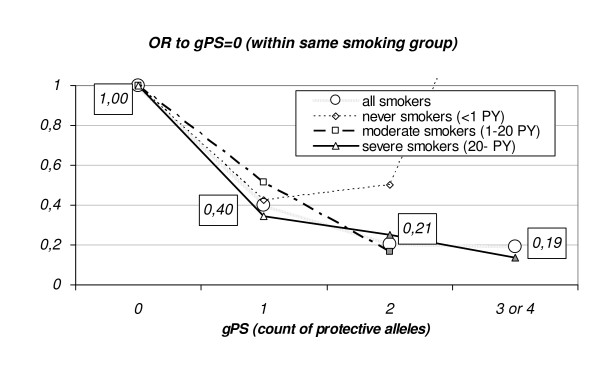
**Change of the protective genetic association for lung cancer with gPS**. gPS: genetic Protection Score:count of protective alleles of GPX1(Pro200Leu) and of EPHX1(His113Tyr). Odds ratio estimates, significant at p < 0.1, are highlighted with filled symbols.

In the presence of one protective allele only (gPS = 1) no differences in the decrease of lung cancer risk compared to gPS = 0 could be found between moderate (OR = 0.5, 95% CI:0.1–3.0), heavy (OR = 0.4, 95%: 0.1–1.3) and never and light smokers (OR = 0.4, 95% CI:0.1–2.0) (see Figure 1 and Table 5). In the presence of two or more protective alleles (gPS > 1) the risk for lung cancer further decreases for moderate and heavy smokers (OR = 0.2, 95% CI:0.1–0.6). For never and light smokers no further risk reduction was observed. However, the subsample of never and light smokers is too small to gain statistical evidence for such a conclusion (only 2 never and light smoking cases have a gPS > 2).

**Table 5:** Genetic association estimates for *GPX1(Pro200Leu) *and *EPHX1(His113Tyr) *accounting for smoking

			Relative chance for LC compared to ...			
			Genetic protected never or light smokers		Genetic protected of the same smoking exposure**.^§^	
SEL: smoking exposure level	gPS*: count of protective alleles	n cases:controls	OR	*95% CI*	OR	95% CI

Never and light smoker	0	8:28	***1.00***	***Reference***	***1.00***	***Reference***
	1	4:39	0.43	0.09 – 1.93	0.43	0.09 – 1.93
	2	2:22	0.50	0.09 – 2.85	0.50	0.09 – 2.85
	3 or 4	2:6	1.91	0.29 – 12.4	1.91	0.29 – 12.4
Moderate smokers	0	15:2	24.7	4.05 – 150	***1.00***	***Reference***
	1	20:8	12.8	3.60 – 45.4	0.51	0.09 – 2.95
	2	9:11	4.1	1.07 – 15.4	0.17	0.03 0.99
	3 or 4	1:0	--		--	
Heavy smokers	0	40:3	74.4	16.2 – 341	***1.00***	***Reference***
	1	57:12	25.7	8.14 – 81.5	0.35	0.09 – 1.31
	2	26:7	18.9	5.15 – 69.2	0.25	0.06 – 1.08
	3 or 4	6:3	10.2	1.78 – 58.2	0.14	0.02 – 0.87

All smokers	0				***1.00***	***Reference***
	1				0.40	0.14 – 1.15
	2.3.4				0.20	0.07 – 0.60

* no. protective alleles of *GPX1(Pro200Leu) *and *EPHX1(His113Tyr)*

** odds ratio. adjusted for sex and age

^§ ^odds ratio of allele-carriers compared to non-carriers within the same smoking group

SEL smoking exposure level: never and light smokers: ≤1 pack years. moderate smokers: 1-<20 pack years. heavysmokers: ≥20 pack years

Please note, even if both DNA-variants independently showed some protective effect for smoking exposed individuals, the risk for lung cancer in the double protected ever smokers (gPS ≥ 3) was significantly higher (OR = 4.8, 95% CI: 1.8–20, p = 0.028) compared to genetically unprotected never and light smokers (gPS = 0).

### NAT2(Gln197Arg)

For the marker ***NAT2(Gln197Arg) ***we did not find an overall association (p_BWS _= 0.252, crude OR_per A-allele _= 1.0, 95% CI: 0.7–1.2). Within the investigated subgroups the BWS-test yielded p-values between 0.163 and 0.495. The only exception were patients with adenocarcinoma (p_BWS _= 0.014).

### GSTP1(A-193C)

For ***GSTP1(A-193C) ***we did not find an overall genetic association (p_BWS _= 0.213, crude OR_per G-allele _= 1.1, 95% CI: 0.8–1.4). In the investigated subgroups the BWS-test yielded p-values between 0.101 and 0.490, with two exceptions. For patients with SCC we achieved a p_BWS _= 0.01. For heavy smokers significance was slightly missed (p_BWS-Test _= 0.089).

For none of the other markers any significant genetic association was observed.

## Discussion

Some chromosomal regions or candidate genes are indicated as associated with lung cancer of any age of onset, additionally and/or interactively to the main risk factor tobacco smoking by several studies yet. For lung cancer with age of onset before age 50 a consistent familial aggregation was observed. The main interest of this study was to investigate the role of some candidate markers for early-onset lung cancer patients.

### GPX1(Pro200Leu)

Antioxidant enzymes like glutathione peroxidase (GPX) are thought to be the primary cellular defence mechanism against reactive oxygen species. The lung epithelium is in particular endangered by exogenous NOx that causes epoxides, aldehydes and peroxides. They react to superoxidradical anion and hydrogen peroxide and in the presence of transition metal ions these continue to react to the aggressive OH radical. These reactive oxygen species (ROS) have the ability to cause massive injury to the cell. They are involved in inflammation processes, peroxidation of membranes which influences their permeability, binding on SH-groups of several enzymes which interferes its activity. Extracellular and intracellular antioxidative defence systems protect the cells from this damage. Extracellular defence is mainly done by small molecular particles like vitamins and small molecular proteins. Intracellular antioxidative defence mostly consists of the anti oxidative enzymes from the glutathion redox cycle (glutathion reductase and glutathion peroxidase). GPX is a tetramerical enzyme with four selenium-atoms bound as selenocystein in the active centre. It is important in the cellular defence against cytotoxic lipid peroxidation products [62]. The catalytic activity of GPX depends on the availability of reduced glutathione as coenzyme and on several endogenous and exogenous influences like genotype and nutrition. Smokers need more protection against these ROS. There is some evidence pointing to a lung cancer protecting role of vitamins C and E. However drinking alcohol is also found to increase the daily demand for vitamins [63,64].

Ratnasinghe et al. [65] found evidence for association with lung cancer for a Pro→Leu (C→T) polymorphism at the amino acid position 200 of the *GPX1*-gene in a sample of Caucasian men aged 50–69 years which currently smoked at least five cigarettes per day. The odds ratio for heterozygotes (CT) was 1.8 (95% CI:1.2–2.8) and 2.3 (95% CI:1.3–3.8) for homozygotes (TT) compared to wildtype (CC) individuals. In a recently published investigation, Raaschou-Nielsen et al. [66] found in general a protective effect of the T-allele, which seems to be stronger in those being diagnosed in the age of 50–60 years (OR_TT vs. CC _= 0.35, 95% CI: 0.2–0.8) than in older patients (OR_TT vs. CC _= 0.8, 95% CI: 0.4–1.6). However, within those smoking more than 20 g tobacco a day the T-allele was found to carry a risk for lung cancer (OR_CT vs. CC _= 1.95*, 95% CI: 1.4–2.6; OR_TT vs. CC _= 1.7*, 95% CI: 1.1–2.7; *per 5 g tobacco a day). Hence, the T-allele increases the risk for lung cancer in smokers. Yang et al. [60] reported for never smokers a non-significant decreased risk for CC-carriers in younger (<50 years, OR = 0.6, 95% CI: 0.3–1.2) and an even stronger and significant decreased risk in older (>80 years, OR = 0.12, 95% CI: 0.02–0.7) patients. However, in older smokers the risk for lung cancer was increased for CC-carriers (OR = 3.3, 95% CI: 1.3–8.4), which is contradictory to the findings of Ratnasinghe et al. and Raaschou-Nielsen et al. The T-allele acts protective against lung cancer in smokers.

While the distributions of genotypes of all mentioned investigations are rather similar within controls, the proportion of the CC-wildtype is much smaller in the case-sample of Ratnasinghe et al. (29%) than in any other case-sample (45% to 61%). With the investigation of Yang et al. [60] we share the focus on early-onset patients. Estimates for OR in younger smokers are not reported by them, but the crude odds ratios of the younger study sample (age <50 years, calculated from table III in Yang et al. [60]) are OR_CT vs. CC _= 0.7 and OR_TT vs. CC _= 0.6 and within the confidence intervals of our estimations (Table 4). Yang et al. constructed hierarchical trees of risk modifying factors performing recursive partitioning (RPART). By this procedure, the sample size of effects in lower hierarchical steps becomes fairly small. They included in their final conclusion a non-significant effect of *GPX1(Pro200Leu) *within never-smokers, but didn't show any effect by *GPX1(Pro200Leu) *within smokers. In contrast to them we could see significant association by *GPX1(Pro200Leu) *for heavy SEL, as for current and former smokers, with ORs from 0.3 to 0.5. We missed significance for never and light and for moderate smokers possibly owing to low sample size. Nevertheless, the estimated effect was of the same size. Hence, T-carriers seem to have some genetic protection against lung cancer within smokers of age 50 years or less. Sensitivity analysis could demonstrate that even under worst conditions for missing genotypes the findings were qualitatively identical and genotyping errors appeared as missing at random. Finally, findings from our sample of early-onset cases are consistent with the previous reports, when restricting samples to age of 50 years or less [60]. However they are in conflict with findings from non-early-onset samples [63,66].

Several factors have yet been identified to modify the activity of GPX. The consumption of fruits and vegetables as well as a supplementation with the trace elements selenium in populations with a low rate of daily intake affect the activity of GPX in human erythrocytes [67]. Serum concentrations of selenium and erythrocyte GPx activity were lower in smokers [68]. Additionally it was reported that alcohol induces lipid peroxidation which might lead to a decrease in GPX activity. Ravn-Haren and colleges could recognize a correlation between alcohol consumption and GPX activity to be modified by the GPX(Pro200Leu) genotype [63,69]. Stronger association between smoking, alcohol intake and lung cancer was seen in carriers of the genotype TT of *GPX1(Pro200Leu) *than in carriers of genotype CC [11].

Thus the observed association between GPX(Pro200Leu) and the risk for lung cancer might be caused by the complex interplay between smoking, nutrition and GPX activity.

#### EPHX1

Microsomal epoxide hydrolase (***EPHX1***) has a putative dual function for enzyme activity which possibly modifies lung cancer risk. On the one hand EPHX1 catalyzes the hydrolysis of epoxides to less reactive substances easier to be solubilised. On the other hand it activates some acrylamine metabolites or polycyclic aromatic hydrocarbons of cigarette smoke into a more carcinogenic form [57]. It is also reported that endotoxin in organic dust induces lung function decline. The strength of such a longitudinal decline is modified by the investigated EPHX1 polymorphism [70].

The activation or inactivation effects of EPHX1 may depend on the specific compounds being metabolized. Changing the structure of the enzyme via polymorphisms in EPHX might have both, protective or promotional effect on developing of lung cancer in smokers. There are two mainly discussed variants of the EPHX gene, one in exon 3 and the other in exon 4. In exon 3 a C has been substituted for a T, resulting in an amino acid exchange at codon 113 (Tyr113His). This amino acid exchange results in a decreasing enzymatic activity (40%–50%) in vitro. In exon 4 a C to A transition causes a histidine to arginine change at codon 139 (His139Arg) with an in vitro increasing enzyme activity (25%) [71].

Two meta-analyses have been recently published investigating the genetic impact of *EPHX1(His113Tyr) *(T→C polymorphism in exon 3) without age constraints and did not find an association with lung cancer (OR = 0.96, 12 studies included [46] and OR = 0.98, 7 studies included [72]). However, Lee et al. [72] reported a significant decrease in lung cancer risk after adjustment for age, sex, smoking and study centre in pooling data of four published and four unpublished case-control studies (OR = 0.7, 95% CI: 0.51–0.96), which is confirmed for a white population in a recently published meta-analysis (5 studies combined, OR = 0.65, 95% CI: 0.44–0.96) [73]. The authors of the first meta-analyses suggested a possible protection for heavy smokers carrying the CC genotype which is in line with our results in young high tar exposed lung cancer patients (OR = 0.8, 94% CI: 0.3–1.9). However, the risk reduction at a moderate level of smoking exposure in our study was estimated even stronger by a point estimate of OR = 0.2, lower than the confidence interval given by Lee et al. [60].

### GPX1(Pro200Leu) and EPHX1(His113Tyr)

In combining both observed protective alleles of both genes we defined gPS, a genetic protection score. We could observe a positive association between the count of protective alleles and the reduction of tobacco smoke induced risk for lung cancer (OR = 0.20, 95% CI: 0.07–0.60).

Within our control group 53% are T-carriers for the *GPX1(Pro200Leu) *variant and 50% are C-carriers for the *EPHX1 *variant. Therefore, we might expect 3 out of 4 individuals of the population to have some genetic protection against lung cancer at younger age. However, the risk raising effect of smoking cigarettes is much stronger. Even under double protection by *GPX1(Pro200Leu) *and *EPHX1(His113Tyr) *the risk of current smokers is at least 4.5-times larger than in unprotected never and light smokers.

In conclusion, our study investigates the association of several candidate genes with lung cancer in the young. Only some of the results from this sample of early-onset lung cancer patients are consistent with previously reported age independent findings or suspicions. However, their role in the developing process is different.

### Some remarks to the study design

We used a candidate gene approach based on the literature lung cancer as a whole. Thus, we can identify no other than previously reported susceptible genes to general age of onset within our young age sample. We also restricted considerations to the most promising marker per gene and did not consider haplotypes within candidate genes. So far, results presented here need to be understood as further investigation of controversial findings. We limited the chance of false positive results by applying a two-step strategy. First we performed two-group-comparisons with BWS-tests, followed by multiple logistic regression modeling for selected markers only.

For four markers the call rate of genotyping is shortly lower than 90%, which results from some suboptimal logistic in the early phase of the study and is not due to genotyping errors.

## Conclusion

Smoking is the most important risk factor for young lung cancer patients. However, this study provides some support for the T-Allel of *GPX1(Pro200Leu) *and the C-Allele of *EPHX1(His113Tyr) *to play a protective role in early onset lung cancer susceptibility.

## Abbreviations

SNP, single nucleotide polymorphism; HWE, Hardy-Weinberg equilibrium; PY, packyears; SEL, smoking exposure level; OR, odds ratio; 95% CI, 95% confidence interval; BWS, Baumgartner-Weiss-Schindler Statistics.

## Competing interests

The author(s) declare that they have no competing interests.

## Authors' contributions

AR and KK participated in quality inspection of the data, performed the statistical analysis and drafted the manuscript. TI and NK managed the storage of the blood samples and performed genotyping. VZ and GB carried out interviews and were responsible for data management. BB and HEW conceived of the study, participated in the design and coordination of the study and drafted the manuscript. EM and GS participated in the design of the study. FK helped to draft the manuscript. All others participated in the recruitment of participants, performed interviews and took blood samples. All authors read and approved the final manuscript.

## Pre-publication history

The pre-publication history for this paper can be accessed here:



## References

[B1] Becker N, Warendorf J (1998). Krebsatlas der Bundesrepublik Deutschland 1981-1990.

[B2] Alberg AJ, Samet JM (2003). Epidemiology of lung cancer. Chest.

[B3] Vineis P, Alavanja M, Buffler P, Fontham E, Franceschi S, Gao YT, Gupta PC, Hackshaw A, Matos E, Samet J, Sitas F, Smith J, Stayner L, Straif K, Thun MJ, Wichmann HE, Wu AH, Zaridze D, Peto R, Doll R (2004). Tobacco and cancer: recent epidemiological evidence. J Natl Cancer Inst.

[B4] Bruske-Hohlfeld I, Mohner M, Pohlabeln H, Ahrens W, Bolm-Audorff U, Kreienbrock L, Kreuzer M, Jahn I, Wichmann HE, Jockel KH (2000). Occupational lung cancer risk for men in Germany: results from a pooled case-control study. Am J Epidemiol.

[B5] Bruske-Hohlfeld I, Mohner M, Ahrens W, Pohlabeln H, Heinrich J, Kreuzer M, Jockel KH, Wichmann HE (1999). Lung cancer risk in male workers occupationally exposed to diesel motor emissions in Germany. Am J Ind Med.

[B6] Jahn I, Ahrens W, Bruske-Hohlfeld I, Kreuzer M, Mohner M, Pohlabeln H, Wichmann HE, Jockel KH (1999). Occupational risk factors for lung cancer in women: results of a case-control study in Germany. Am J Ind Med.

[B7] Pohlabeln H, Boffetta P, Ahrens W, Merletti F, Agudo A, Benhamou E, Benhamou S, Bruske-Hohlfeld I, Ferro G, Fortes C, Kreuzer M, Mendes A, Nyberg F, Pershagen G, Saracci R, Schmid G, Siemiatycki J, Simonato L, Whitley E, Wichmann HE, Winck C, Zambon P, Jockel KH (2000). Occupational risks for lung cancer among nonsmokers. Epidemiology.

[B8] Darby S, Hill D, Auvinen A, Barros-Dios JM, Baysson H, Bochicchio F, Deo H, Falk R, Forastiere F, Hakama M, Heid I, Kreienbrock L, Kreuzer M, Lagarde F, Makelainen I, Muirhead C, Oberaigner W, Pershagen G, Ruano-Ravina A, Ruosteenoja E, Rosario AS, Tirmarche M, Tomasek L, Whitley E, Wichmann HE, Doll R (2005). Radon in homes and risk of lung cancer: collaborative analysis of individual data from 13 European case-control studies. BMJ.

[B9] Wichmann HE, Rosario AS, Heid IM, Kreuzer M, Heinrich J, Kreienbrock L (2005). Increased lung cancer risk due to residential radon in a pooled and extended analysis of studies in Germany. Health Phys.

[B10] Cohen AJ, Ross AH, Ostro B, Pandey KD, Krzyzanowski M, Kunzli N, Gutschmidt K, Pope A, Romieu I, Samet JM, Smith K (2005). The global burden of disease due to outdoor air pollution. J Toxicol Environ Health A.

[B11] Boffetta P, Agudo A, Ahrens W, Benhamou E, Benhamou S, Darby SC, Ferro G, Fortes C, Gonzalez CA, Jockel KH, Krauss M, Kreienbrock L, Kreuzer M, Mendes A, Merletti F, Nyberg F, Pershagen G, Pohlabeln H, Riboli E, Schmid G, Simonato L, Tredaniel J, Whitley E, Wichmann HE, Winck C, Zambon P, Saracci R (1998). Multicenter case-control study of exposure to environmental tobacco smoke and lung cancer in Europe. J Natl Cancer Inst.

[B12] Brennan P, Buffler PA, Reynolds P, Wu AH, Wichmann HE, Agudo A, Pershagen G, Jockel KH, Benhamou S, Greenberg RS, Merletti F, Winck C, Fontham ET, Kreuzer M, Darby SC, Forastiere F, Simonato L, Boffetta P (2004). Secondhand smoke exposure in adulthood and risk of lung cancer among never smokers: a pooled analysis of two large studies. Int J Cancer.

[B13] Kreuzer M, Krauss M, Kreienbrock L, Jockel KH, Wichmann HE (2000). Environmental tobacco smoke and lung cancer: a case-control study in Germany. Am J Epidemiol.

[B14] Brennan P, Butler J, Agudo A, Benhamou S, Darby S, Fortes C, Jockel KH, Kreuzer M, Nyberg F, Pohlabeln H, Saracci R, Wichman HE, Boffetta P (2000). Joint effect of diet and environmental tobacco smoke on risk of lung cancer among nonsmokers. J Natl Cancer Inst.

[B15] Alexandrov K, Cascorbi I, Rojas M, Bouvier G, Kriek E, Bartsch H (2002). CYP1A1 and GSTM1 genotypes affect benzo[a]pyrene DNA adducts in smokers' lung: comparison with aromatic/hydrophobic adduct formation. Carcinogenesis.

[B16] Bourke W, Milstein D, Giura R, Donghi M, Luisetti M, Rubin AH, Smith LJ (1992). Lung cancer in young adults. Chest.

[B17] Green LS, Fortoul TI, Ponciano G, Robles C, Rivero O (1993). Bronchogenic cancer in patients under 40 years old. The experience of a Latin American country. Chest.

[B18] Icard P, Regnard JF, de Napoli S, Rojas-Miranda A, Dartevelle P, Levasseur P (1992). Primary lung cancer in young patients: a study of 82 surgically treated patients. Ann Thorac Surg.

[B19] Jubelirer SJ, Wilson RA (1991). Lung cancer in patients younger than 40 years of age. Cancer.

[B20] Kreuzer M, Kreienbrock L, Muller KM, Gerken M, Wichmann E (1999). Histologic types of lung carcinoma and age at onset. Cancer.

[B21] Larrieu AJ, Jamieson WR, Nelems JM, Fowler R, Yamamoto B, Leriche J, Murray N (1985). Carcinoma of the lung in patients under 40 years of age. Am J Surg.

[B22] Rocha MP, Fraire AE, Guntupalli KK, Greenberg SD (1994). Lung cancer in the young. Cancer Detect Prev.

[B23] Roviaro GC, Varoli F, Zannini P, Fascianella A, Pezzuoli G (1985). Lung cancer in the young. Chest.

[B24] Tsugane S, Watanabe S, Sugimura H, Arimoto H, Shimosato Y, Suemasu K (1987). Smoking, occupation and family history in lung cancer patients under fifty years of age. Jpn J Clin Oncol.

[B25] Kreuzer M, Pohlabeln H, Ahrens W, Kreienbrock L, Bruske-Hohlfeld I, Jockel KH, Wichmann HE (1999). Occupational risk factors for lung cancer among young men. Scand J Work Environ Health.

[B26] Kreuzer M, Wichmann HE (2001). Lung cancer in young females. Eur Respir J.

[B27] Kreuzer M, Kreienbrock L, Gerken M, Heinrich J, Bruske-Hohlfeld I, Muller KM, Wichmann HE (1998). Risk factors for lung cancer in young adults. Am J Epidemiol.

[B28] Ambrosone CB, Rao U, Michalek AM, Cummings KM, Mettlin CJ (1993). Lung cancer histologic types and family history of cancer. Analysis of histologic subtypes of 872 patients with primary lung cancer. Cancer.

[B29] Horwitz RI, Smaldone LF, Viscoli CM (1988). An ecogenetic hypothesis for lung cancer in women. Arch Intern Med.

[B30] Jin YT, He XZ (1993). [Analysis of familial aggregation of lung cancer in Xuanwei]. Zhonghua Yu Fang Yi Xue Za Zhi.

[B31] McDuffie HH, Klaassen DJ, Dosman JA (1989). Characteristics of patients with primary lung cancer diagnosed at age of 50 years or younger. Chest.

[B32] Ooi WL, Elston RC, Chen VW, Bailey-Wilson JE, Rothschild H (1986). Increased familial risk for lung cancer. J Natl Cancer Inst.

[B33] Osann KE (1991). Lung cancer in women: the importance of smoking, family history of cancer, and medical history of respiratory disease. Cancer Res.

[B34] Radzikowska E, Rowinska-Zakrzewska E (1994). [Familial aggregation of lung cancer in families of patients with lung cancer]. Pneumonol Alergol Pol.

[B35] Samet JM, Humble CG, Pathak DR (1986). Personal and family history of respiratory disease and lung cancer risk. Am Rev Respir Dis.

[B36] Shaw GL, Falk RT, Pickle LW, Mason TJ, Buffler PA (1991). Lung cancer risk associated with cancer in relatives. J Clin Epidemiol.

[B37] TOKUHATA GK, LILIENFELD AM (1963). Familial aggregation of lung cancer in humans. J Natl Cancer Inst.

[B38] Bromen K, Pohlabeln H, Jahn I, Ahrens W, Jockel KH (2000). Aggregation of lung cancer in families: results from a population-based case-control study in Germany. Am J Epidemiol.

[B39] Schwartz AG, Yang P, Swanson GM (1996). Familial risk of lung cancer among nonsmokers and their relatives. Am J Epidemiol.

[B40] Yang P, Schwartz AG, McAllister AE, Swanson GM, Aston CE (1999). Lung cancer risk in families of nonsmoking probands: heterogeneity by age at diagnosis. Genet Epidemiol.

[B41] Lichtenstein P, Holm NV, Verkasalo PK, Iliadou A, Kaprio J, Koskenvuo M, Pukkala E, Skytthe A, Hemminki K (2000). Environmental and heritable factors in the causation of cancer--analyses of cohorts of twins from Sweden, Denmark, and Finland. N Engl J Med.

[B42] Braun MM, Caporaso NE, Page WF, Hoover RN (1994). Genetic component of lung cancer: cohort study of twins. Lancet.

[B43] Bailey-Wilson JE, Amos CI, Pinney SM, Petersen GM, de Andrade M, Wiest JS, Fain P, Schwartz AG, You M, Franklin W, Klein C, Gazdar A, Rothschild H, Mandal D, Coons T, Slusser J, Lee J, Gaba C, Kupert E, Perez A, Zhou X, Zeng D, Liu Q, Zhang Q, Seminara D, Minna J, Anderson MW (2004). A major lung cancer susceptibility locus maps to chromosome 6q23-25. Am J Hum Genet.

[B44] Bouchardy C, Benhamou S, Jourenkova N, Dayer P, Hirvonen A (2001). Metabolic genetic polymorphisms and susceptibility to lung cancer. Lung Cancer.

[B45] Spivack SD, Fasco MJ, Walker VE, Kaminsky LS (1997). The molecular epidemiology of lung cancer. Crit Rev Toxicol.

[B46] Kiyohara C, Otsu A, Shirakawa T, Fukuda S, Hopkin JM (2002). Genetic polymorphisms and lung cancer susceptibility: a review. Lung Cancer.

[B47] Goode EL, Ulrich CM, Potter JD (2002). Polymorphisms in DNA repair genes and associations with cancer risk. Cancer Epidemiol Biomarkers Prev.

[B48] Hou SM, Falt S, Angelini S, Yang K, Nyberg F, Lambert B, Hemminki K (2002). The XPD variant alleles are associated with increased aromatic DNA adduct level and lung cancer risk. Carcinogenesis.

[B49] Zhou W, Liu G, Miller DP, Thurston SW, Xu LL, Wain JC, Lynch TJ, Su L, Christiani DC (2002). Gene-environment interaction for the ERCC2 polymorphisms and cumulative cigarette smoking exposure in lung cancer. Cancer Res.

[B50] Weidinger S, Klopp N, Wagenpfeil S, Rummler L, Schedel M, Kabesch M, Schafer T, Darsow U, Jakob T, Behrendt H, Wichmann HE, Ring J, Illig T (2004). Association of a STAT 6 haplotype with elevated serum IgE levels in a population based cohort of white adults. J Med Genet.

[B51] Steffens M, Lamina C, Illig T, Bettecken T, Vogler R, Entz P, Suk EK, Toliat MR, Klopp N, Caliebe A, Konig IR, Kohler K, Ludemann J, Diaz LA, Fimmers R, Lichtner P, Ziegler A, Wolf A, Krawczak M, Nurnberg P, Hampe J, Schreiber S, Meitinger T, Wichmann HE, Roeder K, Wienker TF, Baur MP (2006). SNP-based analysis of genetic substructure in the German population. Hum Hered.

[B52] Latza U, Hoffmann W, Terschüren C, Chang-claude C, Kreuzer M, Schaffrath Rosario A, Kropp S, Stang A, Ahrens W, Lampert T (2005). Erhebung, Quantifizierung und Analyse der Rauchexposition in epidemiologischen Studien.

[B53] Bernaards CM, Twisk JW, Snel J, Van Mechelen W, Kemper HC (2001). Is calculating pack-years retrospectively a valid method to estimate life-time tobacco smoking? A comparison between prospectively calculated pack-years and retrospectively calculated pack-years. Addiction.

[B54] Weir BS (1996). Genetic data analysis II
methods for discrete population genetic data.

[B55] Neuhauser M (2002). Exact tests for the analysis of case-control studies of genetic markers. Hum Hered.

[B56] (2004). Mikrozensus 2003 - Fragen zur Gesundheit.

[B57] Gouaze V, Andrieu-Abadie N, Cuvillier O, Malagarie-Cazenave S, Frisach MF, Mirault ME, Levade T (2002). Glutathione peroxidase-1 protects from CD95-induced apoptosis. J Biol Chem.

[B58] Ravn-Haren G, Olsen A, Tjonneland A, Dragsted LO, Nexo BA, Wallin H, Overvad K, Raaschou-Nielsen O, Vogel U (2006). Associations between GPX1 Pro198Leu polymorphism, erythrocyte GPX activity, alcohol consumption and breast cancer risk in a prospective cohort study. Carcinogenesis.

[B59] Fabricius P, Lange P (2003). Diet and lung cancer. Monaldi Arch Chest Dis.

[B60] Ratnasinghe D, Tangrea JA, Andersen MR, Barrett MJ, Virtamo J, Taylor PR, Albanes D (2000). Glutathione peroxidase codon 198 polymorphism variant increases lung cancer risk. Cancer Res.

[B61] Raaschou-Nielsen O, Sorensen M, Hansen RD, Frederiksen K, Tjonneland A, Overvad K, Vogel U (2007). GPX1 Pro198Leu polymorphism, interactions with smoking and alcohol consumption, and risk for lung cancer. Cancer Lett.

[B62] Yang P, Bamlet WR, Ebbert JO, Taylor WR, de Andrade M (2004). Glutathione pathway genes and lung cancer risk in young and old populations. Carcinogenesis.

[B63] Silvera SA, Rohan TE (2007). Trace elements and cancer risk: a review of the epidemiologic evidence. Cancer Causes Control.

[B64] Northrop-Clewes CA, Thurnham DI (2007). Monitoring micronutrients in cigarette smokers. Clin Chim Acta.

[B65] Hu YJ, Diamond AM (2003). Role of glutathione peroxidase 1 in breast cancer: loss of heterozygosity and allelic differences in the response to selenium. Cancer Res.

[B66] Garte S, Gaspari L, Alexandrie AK, Ambrosone C, Autrup H, Autrup JL, Baranova H, Bathum L, Benhamou S, Boffetta P, Bouchardy C, Breskvar K, Brockmoller J, Cascorbi I, Clapper ML, Coutelle C, Daly A, Dell'Omo M, Dolzan V, Dresler CM, Fryer A, Haugen A, Hein DW, Hildesheim A, Hirvonen A, Hsieh LL, Ingelman-Sundberg M, Kalina I, Kang D, Kihara M, Kiyohara C, Kremers P, Lazarus P, Le Marchand L, Lechner MC, van Lieshout EM, London S, Manni JJ, Maugard CM, Morita S, Nazar-Stewart V, Noda K, Oda Y, Parl FF, Pastorelli R, Persson I, Peters WH, Rannug A, Rebbeck T, Risch A, Roelandt L, Romkes M, Ryberg D, Salagovic J, Schoket B, Seidegard J, Shields PG, Sim E, Sinnet D, Strange RC, Stucker I, Sugimura H, To-Figueras J, Vineis P, Yu MC, Taioli E (2001). Metabolic gene polymorphism frequencies in control populations. Cancer Epidemiol Biomarkers Prev.

[B67] Hang J, Zhou W, Wang X, Zhang H, Sun B, Dai H, Su L, Christiani DC (2005). Microsomal epoxide hydrolase, endotoxin, and lung function decline in cotton textile workers. Am J Respir Crit Care Med.

[B68] Hassett C, Aicher L, Sidhu JS, Omiecinski CJ (1994). Human microsomal epoxide hydrolase: genetic polymorphism and functional expression in vitro of amino acid variants. Hum Mol Genet.

[B69] Lee WJ, Brennan P, Boffetta P, London SJ, Benhamou S, Rannug A, To-Figueras J, Ingelman-Sundberg M, Shields P, Gaspari L, Taioli E (2002). Microsomal epoxide hydrolase polymorphisms and lung cancer risk: a quantitative review. Biomarkers.

[B70] Kiyohara C, Yoshimasu K, Takayama K, Nakanishi Y (2006). EPHX1 polymorphisms and the risk of lung cancer: a HuGE review. Epidemiology.

[B71] AS J (2002). Exploring the functional plasticity of human gluthatione transferases.

[B72] Lunn RM, Langlois RG, Hsieh LL, Thompson CL, Bell DA (1999). XRCC1 polymorphisms: effects on aflatoxin B1-DNA adducts and glycophorin A variant frequency. Cancer Res.

[B73] Biros E, Kalina I, Kohut A, Bogyiova E, Salagovic J, Sulla I (2002). Allelic and haplotype frequencies of the p53 polymorphisms in brain tumor patients. Physiol Res.

